# Comparative Hippocampal Synaptic Proteomes of Rodents and Primates: Differences in Neuroplasticity-Related Proteins

**DOI:** 10.3389/fnmol.2018.00364

**Published:** 2018-10-02

**Authors:** Frank Koopmans, Nikhil J. Pandya, Sigrid K. Franke, Ingrid H.C.M.H. Phillippens, Iryna Paliukhovich, Ka Wan Li, August B. Smit

**Affiliations:** ^1^Department of Molecular and Cellular Neurobiology, Center for Neurogenomics and Cognitive Research, Amsterdam Neuroscience, Vrije Universiteit Amsterdam, Amsterdam, Netherlands; ^2^Biomedical Primate Research Centre, Rijswijk, Netherlands

**Keywords:** synapse, hippocampus, species, neuroplasticity, proteomics, SWATH-MS

## Abstract

Key to the human brain’s unique capacities are a myriad of neural cell types, specialized molecular expression signatures, and complex patterns of neuronal connectivity. Neurons in the human brain communicate via well over a quadrillion synapses. Their specific contribution might be key to the dynamic activity patterns that underlie primate-specific cognitive function. Recently, functional differences were described in transmission capabilities of human and rat synapses. To test whether unique expression signatures of synaptic proteins are at the basis of this, we performed a quantitative analysis of the hippocampal synaptic proteome of four mammalian species, two primates, human and marmoset, and two rodents, rat and mouse. Abundance differences down to 1.15-fold at an FDR-corrected *p*-value of 0.005 were reliably detected using SWATH mass spectrometry. The high measurement accuracy of SWATH allowed the detection of a large group of differentially expressed proteins between individual species and rodent vs. primate. Differentially expressed proteins between rodent and primate were found highly enriched for plasticity-related proteins.

## Introduction

The human brain’s unique cognitive capacity is probably not only derived from its absolute or relative size, or even its number of neurons and glia, but also involves increased diversity of molecular expression signatures, neural cell types, and typical spatial and temporal development leading to expanded and/or more complex patterns of neuronal connectivity. Humans have specialized neuronal connections and a myriad of neuronal cell types which communicate via an estimated well over quadrillion synapses in the human central nervous system (Silbereis et al., 2016). Specific synaptic connections form the core components of neural circuits and networks, collectively referred to as the connectome (van den Heuvel et al., 2016), and their contribution might be key to the dynamic activity patterns that underlie species-specific cognitive function (Mesulam, 2000; Markov et al., 2013; van den Heuvel et al., 2016).

The mammalian brain is capable of processing information in parallel, at high speed and in a highly adaptive manner. These features are largely governed by fast transmission in highly plastic excitatory glutamatergic synapses. Over the years, many proteins of the synapse, their subcellular enrichment, and molecular organization into functional entities has become apparent (Chua, 2014; Pandya et al., 2017). Examples of these functional entities are the resident proteins of the presynaptic vesicle, proteins of the fusion, and release machinery (Jahn and Fasshauer, 2012) or smaller functional units, such as those associated to synaptic calcium channels (Muller et al., 2010) or glutamate receptors (Schwenk et al., 2012; Chen et al., 2014). Basic synaptic features, such as vesicle release and receptor-mediated signal transduction, are largely carried by evolutionary strongly sequence-conserved proteins (Bayes et al., 2017). Previous studies have indicated changes in the components of the glutamatergic signaling pathway during primate brain evolution in terms of gene expression, protein expression, and promoter sequence changes (Muntane et al., 2015). An outstanding question however is whether the levels of synaptic proteins that underlie the stoichiometries of protein-protein interactions and govern their function in molecular assemblies, have remained conserved.

Intriguingly, recent findings show that human and mouse synapses do not differ in aspects of basic transmission, but drastically differ functionally in the capability to confer high frequency signals (Testa-Silva et al., 2014). Features of synaptic plasticity may also be differently organized, such as observed in spike time dependent plasticity comparing human and rat synapses (Testa-Silva et al., 2010).

Therefore, in this study we investigated the expression signature of the mammalian hippocampal synaptic proteome of rodents; mouse and rat, and primates; marmoset and human. First, we investigated how the expression of synaptic proteins has evolved between these species. This is relevant as alterations in synaptic function are carried by the synaptic protein interaction network and are likely caused by the underlying changes in expression of proteins. Secondly, we investigated whether evolutionary dictated expression differences might relate to plasticity features of synapses. The hippocampus was selected for its well-known role in learning and memory, and the well-described occurrence of correlated synaptic plasticity features, apparent in long-term potentiation (LTP) or long-term depression (LTD) (Cooper, 2003). Of technical importance, the hippocampus is a neuro-anatomically distinct structure that can be dissected in a reproducible way from the brain of different mammalian species, giving credence to the comparative analysis of this study (Spijker, 2011).

Proteomics analysis was performed using SWATH mass spectrometry (a type of Data Independent Acquisition, DIA), which has been developed to allow for a complete recording of all fragment ions of all (detectable) peptides in a given sample (Gillet et al., 2012). Key advantages of SWATH are a strong reduction in missing values and lowered Coefficient of Variation between (replicate) measurements compared to traditional shotgun proteomics (Bruderer et al., 2015; Koopmans et al., 2018). SWATH analysis enabled a sensitive comparative analysis of the hippocampal synaptic proteome of four species. We first delineated bona fide pre- and postsynaptic proteins and determined their abundance in known protein complexes. In these, we discriminated synaptic substructures and functionalities, such as elements of the postsynapse, e.g., the postsynaptic density, and the presynaptic release machinery. This analysis revealed that within the inter-species conserved synaptic proteome distinct ratiometric differences are apparent, which are species and/or order specific. Proteins involved in this are enriched for synaptic plasticity function suggesting that selective expression differences subserve plasticity and cognitive function.

## Materials and Methods

### Animal and Human Tissue Use

The use of rodent brain material was approved by the animal ethics committee of the Vrije Universiteit Amsterdam. The use of marmoset brain material was approved by the Biomedical Primate Research Centre (BPRC) ethics committee before the start of experiments, according to Dutch law. Human hippocampus brain samples with donor consent were obtained from, and used according to the guidelines of, the Dutch Brain Bank.

### Dissection of Hippocampal Tissue

Whole hippocampus was dissected from male mouse brain (C57B6/J; Charles River, France) and from male rats (Wistar; Harlan, Netherlands) (**Table 1**). Hippocampus of the common marmoset (Callithrix jacchus; Biomedical Primate Research Centre, Rijswijk, The Netherlands) was taken between Bregma -2.00 and +1.50. Human postmortem brain tissue from individuals without neurological disorders was obtained from the Netherlands Brain Bank (NBB), Netherlands Institute for Neuroscience, Amsterdam (**Table 1**). All brain tissue has been collected from donors with written informed consent for brain autopsy, and approval to use this tissue for research purposes has been obtained by the NBB. Approximately 50 mg of tissue was isolated from fresh frozen human hippocampal brain tissue by making slices of 20 μm using a cryostat. Brain slices include all hippocampal subregions and a small part of the temporal lobe. The tissue was collected in pre-weighed and cooled Eppendorf tubes. All tissues dissected were stored at -80°C until later use. Postmortem delay times of human tissue 4–7 h, marmoset < 20 min., rodents < 10 min.

**Table 1:** Overview of all samples used in this study.

Sample	Sex	Age at Dissection	Tissue wet weight (mg)
Mouse-1	M	50 Weeks	47
Mouse-2	M	50 Weeks	43
Mouse-3	M	50 Weeks	35
Mouse-4	M	50 Weeks	46
Mouse-5	M	50 Weeks	45
Rat-1	M	50 Weeks	160
Rat-2	M	50 Weeks	159
Rat-3	M	50 Weeks	148
Rat-4	M	50 Weeks	174
Rat-5	M	50 Weeks	175
Marmoset-1	F	5,6 years	58
Marmoset-2	M	3,2 years	88
Marmoset-3	M	7 years	44
Marmoset-4	M	3,1 years	58
Marmoset-5	F	3,2 years	45
Marmoset-6	F	2,7 years	65
Human-1	F	50–55 years	62
Human-2	M	56–60 years	45
Human-3	M	50–55 years	42
Human-4	M	46–50 years	52
Human-5	M	50–55 years	48


### Synaptosome Isolation

Synaptosomes were isolated as described previously (Pandya et al., 2017). To correct for differences in amount of input material between different species, we used 50 μl of homogenization buffer per mg of tissue to ensure that the homogenization conditions were identical between species. Post-homogenization, the samples were spun at 1000 × g for 10 min. and the supernatant was loaded on a sucrose gradient of 1.2/0.85M followed by ultracentrifugation at 100,000 × g for 2 h. Synaptosomes were collected at the interface of 1.2/0.85M, mixed with 5 ml homogenization buffer and centrifuged at 20,000 × g for 30 min to obtain the synaptosomal pellets, which were stored at -80°C prior to the FASP procedure.

### FASP In-Solution Digestion of Proteins

Samples were digested using the FASP in-solution digestion protocol (Wisniewski et al., 2009) with some modifications according to (Pandya et al., 2017). 10 μg of synaptosomes from each sample was incubated with 75 μl 2% SDS 1 uL 50 mM Tris (2-carboxyethyl)phosphine (TCEP) reducing agent at 55°C for 1 h at 900 rpm. Next, the sample was incubated with 0.5 uL 200 mM methyl methanethiosulfonate (MMTS) for 15 min at RT with shaking, after which samples were transferred to YM-30 filters (Microcon^®^, Millipore) after addition of 200 μl 8 M Urea in Tris buffer (pH 8.8). Samples were washed with 8M Urea in Tris buffer 5 times by spinning at 14,000 × g for 10 min each followed by 4 washes with 50 mM NH_4_HCO_3_. Finally, the samples were incubated with 100 μl of Trypsin overnight in a humidified chamber at 37°C for 12 h. Digested peptides were eluted from the filter with 0.1% acetic acid. The peptides in solution were dried using a speedvac and stored at -20°C prior to LC-MS analysis.

### SCX Fractionation

Peptides obtained from 100 μg of synaptosomes following the FASP procedure were fractionated using strong cation-exchange chromatography as described previously (Gonzalez-Lozano et al., 2016). Peptide samples were loaded onto a 4.6 × 100 mm polysulfoethyl A column (PolyLC) and separated using a non-linear gradient of 60 min at 200 μl/min solvent A (10 mM KH_2_PO_4_, 20% acetonitrile, pH 2.9) and solvent B (solvent A+500 mM KCl). From 0–10 min, flow of 100% solvent A, from 10–35 min solvent B was increased to 65%. In the next 5 min, solvent B was increased to 100% followed by 8 min of wash with 100% solvent A. In total 40 fractions of 200 μl each were collected. Fractions were pooled in the following manner: 16–20 min (Fraction 1), 21–22 min (Fraction 2), 23–24 min (Fraction 3), 25–26 min (Fraction 4), 27–28 min (Fraction 5), 28–38 min (Fraction 6). Fraction 6 was desalted using Oasis column prior to LC-MS/MS DDA analysis.

### Micro-LC and Data-dependent Data Acquisition Mass Spectrometry of SCX Fractions

Peptides were analyzed by micro LC MS/MS using an Ultimate 3000 LC system (Dionex, Thermo Scientific) coupled to the TripleTOF 5,600 mass spectrometer (Sciex). Peptides were trapped on a 5 mm Pepmap 100 C18 column (300 μm i.d., 5 μm particle size, Dionex) and fractionated on a 200 mm Alltima C18 column (300 μm i.d., 3 μm particle size). The acetonitrile concentration in the mobile phase was increased from 5 to 18% in 88 min, to 25% at 98 min, 40% at 108 min and to 90% in 2 min, at a flow rate of 5 μL/min. The eluted peptides were electro-sprayed into the TripleTOF MS. The micro-spray needle voltage was set to 5,500V. The mass spectrometer was operated in a data-dependent mode with a single MS full scan (m/z 350-1250, 150 msec) followed by a top 25 MS/MS (m/z 200–1800, 150 msec) at high sensitivity mode in UNIT resolution, precursor ion > 150 counts/s, charge state from +2 to +5) with an exclusion time of 16 sec once the peptide was fragmented. Ions were fragmented in the collision cell using rolling collision energy, and a spread energy of 5 eV.

### Micro-LC and SWATH Mass Spectrometry

The conditions used for LC in SWATH MS-based experiments were the same as those of the DDA experiments. SWATH experiments consisted of a parent ion scan of 150 msec followed by SWATH window of 8 Da with scan time of 80 msec, and stepped through the mass range between 450–770 m/z. The total cycle time was about 3.2 sec, which yielded in general 9–10 measurement points across a typical peptide with an elution time of 30 sec. The collision energy for each window was determined based on the appropriate collision energy for a 2+ ion, centered upon the window with a spread of 15 eV. The mass spectrometry proteomics data have been deposited to the ProteomeXchange Consortium via the PRIDE (Vizcaino et al., 2016) partner repository with the dataset identifier PXD009251.

### Analysis of Data-dependent Acquisition Mass Spectrometry

LC-MS data measured in DDA mode was analyzed using MaxQuant 1.5.2.8 (Cox and Mann, 2008). An initial search using a 0.07 Da peptide mass tolerance was followed by a correction of systematic mass errors. The calibrated data was then subjected to the main search with a 0.006 Da peptide mass tolerance. The minimum peptide length was set to 6, with at most two miss-cleavages allowed. Methionine oxidation and N-terminal acetylation were set as variable modifications with cysteine beta-methylthiolation set as fixed modification.

MS/MS spectra were searched against the human proteome using the UniProt (release February 2016) FASTA database that includes both reviewed (Swiss-Prot) and unreviewed (TrEMBL) records and both canonical and isoform sequences. The Biognosys iRT FASTA database was also included in order to ensure that iRT peptides were included in the search results, as these were used to normalize retention times in downstream analysis. For both peptide and protein identification a false discovery rate of 0.01 was set. The computation of iBAQ (Schwanhausser et al., 2011) in-silico estimated absolute protein abundances (by dividing the protein intensity by the number of theoretically observable tryptic peptides) was enabled.

### SWATH Data Extraction and Analysis

A spectral library was made from the MaxQuant analysis of DDA data using Spectronaut 6.0.6880.14 (Bruderer et al., 2015). The Q-value threshold for peptides imported from the MaxQuant *msms.txt* output table was set to 0.01, all other settings were left to default.

Next, Spectronaut was used to extract peptide abundances from the raw SWATH data. The retention time prediction type was set to dynamic iRT and profiling peak refinement was enabled. Finally, across-run normalization based on total peak areas was performed by Spectronaut.

Peptide abundances were exported as a Spectronaut report and further processed using the R language for statistical computation (R Core Team, 2014), in which we considered each unique precursor as a peptide (e.g., the same peptide sequence observed with distinct modifications or charge was considered a distinct peptide).

Spectronaut’s fragment group Q-values were used to discriminate high confidence peptides. For the validation of SWATH capabilities using three technical replicates, all peptides with a Q-value higher than 10^-3^ in any sample were removed.

For the pairwise comparison between (groups of) species, we used the subset of peptides that are present in all species and quantified with high confidence in either group. The former condition was reached by comparing the *in-silico* digestion of the respective FASTA database of all species. The latter is formalized as having a Q-value smaller than, or equal to, 10^-4^ over all samples (while allowing for one outlier within each species) in either group.

Protein abundances were computed by summation of the normalized peak area of their respective peptides. Peptides that map ambiguously to multiple genes in the spectral library were discarded. Finally, the protein abundance matrix was Loess normalized using the *normalizeCyclicLoess* function from the limma R package (Smyth et al., 2005), which was set to ‘fast’ and iterations were set to 10.

Synaptic plasticity proteins listed in **Supplementary Table S2** of (Rao-Ruiz et al., 2015) were used for the statistical analysis of synaptic plasticity within our SWATH data. Overlap between this set and SWATH quantified proteins from this study can be found in the last column of **Supplementary Tables S2**–**S4**.

### Differential Abundance Analysis

Differential abundance analysis between (groups of) species was performed on log transformed protein abundances. Empirical Bayes moderated t-statistics with multiple testing correction by False Discovery Rate (FDR), as implemented by the *eBayes* and *topTable* functions from the limma R package, was used. An FDR adjusted *p*-value threshold of 0.005 was used to discriminate proteins of interest after differential abundance analysis. Gene Ontology enrichment tests were performed using the PANTHER Overrepresentation Test (version 13.1) with the total set of SWATH quantified proteins as the background set (Mi et al., 2017).

## Results

Brain tissue was obtained taking age and gender matching into account. Human subjects were on average 53.1 ± 3.7 years of age and animal groups were chosen in line with this, e.g., with mouse and rat 50 weeks of age and marmoset on average 4 years of age (**Table 1**). Whole hippocampus from mouse, rat and marmoset was dissected. To enable direct comparison of the rodent and marmoset hippocampus samples with the much larger human hippocampus, we made 20 μm cross section slices of human hippocampus that included all sub-regions. Synaptosome fractions were prepared from independent biological replicates (*n* = 6 for marmoset and *n* = 5 for the other species), as described previously (Pandya et al., 2017).

### Spectral Library for SWATH Analysis

For SWATH analysis, a spectral library that contains a fingerprint (e.g., m/z and retention time) of each peptide is used for targeted data extraction to obtain peptide abundancy values. A key challenge in building the spectral library is the inclusion of low abundant proteins as the probability of protein detection in discovery proteomics is correlated with their abundance. Therefore, an extensive spectral library was prepared from the synaptosome fraction of each species using Data Dependent Analysis (DDA) proteomics and extensive SCX fractionation to optimize coverage of low abundant proteins.

Since the SWATH approach compares abundance levels of the exact same peptide between samples, we used the DDA data to create a spectral library of detectable peptides in the human synaptic proteome. This was later used to compare abundance values of human synaptic proteins among species. The rationale for using DDA of all species to identify the human synaptic proteome, and not only human samples, lies in the assumption that protein levels may vary among species (for instance, proteins more abundant in mouse have better detection probability in mouse). Using MaxQuant, 681626 MS/MS spectra were searched against the UniProt human proteome, resulting in the identification of 29710 unique peptide sequences and 3937 protein groups. 166 protein groups that contained proteins from different genes were considered ambiguous and removed from the dataset. The dynamic range of synaptic protein levels was investigated using iBAQ abundances (an *in-silico* estimation of absolute protein abundance using peptide intensity values) obtained from the DDA data. Protein groups that mapped to the same gene were merged by summation of their iBAQ abundances, yielding a final dataset containing absolute abundance levels for in total 3630 proteins (**Supplementary Table S1**).

**FIGURE 1:**
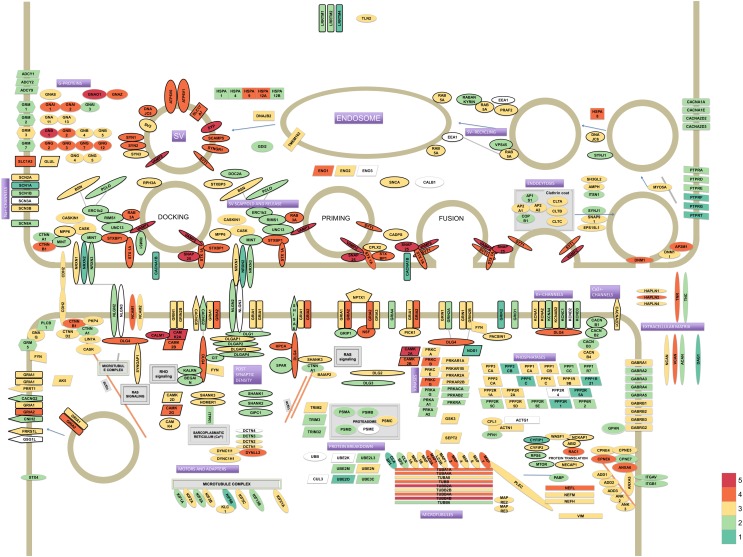
Model of the synaptic proteome featuring 336 proteins selected from literature. Protein colors reflect their estimated abundances in the synaptosome fraction of human hippocampus. The iBAQ values (which were approximately log-normal distributed) were log10 transformed to improve visualization of differences. The color legend for depicted log10 iBAQ values is shown in the bottom-right; low abundant proteins are depicted in green, medium in yellow and high abundant proteins are shown in red.

From the thousands of identified proteins we confidently assigned 336 proteins to the pre- or postsynapse, or are synaptic without a definite pre- or postsynaptic localization (**Figure 1**). These core synaptic proteins were derived from data from previous analyses (Chua et al., 2010; Weingarten et al., 2014; Wilhelm et al., 2014). These 336 proteins span close to 6 orders of magnitude difference in iBAQ abundance (**Supplementary Table S1**). In particular, presynaptic proteins belonging to the synaptic vesicle and those serving the actual vesicle to membrane fusion event were highly abundant. Much lower abundant were proteins typical of the postsynapse, such as postsynaptic receptors and their auxiliary subunits.

### SWATH Workflow and Quality Control

The SWATH mass spectrometry data were processed in Spectronaut using our synapse spectral library and the resulting qualitative and quantitative peptide data were processed using the R language for statistical computation.

We first assessed the performance of the SWATH proteomics pipeline by determining the technical variation observed in a triplicate measurement of a single mouse synaptosome preparation. Using only peptides identified in the mouse synaptosome samples from the spectral library that were quantified confidently in all three SWATH technical replicates, we calculated protein abundances and applied Loess normalization. The median Coefficient of Variation (CoV) for 5144 peptides was 4% (**Supplementary Figure S1A**) and the median CoV of 1831 quantified proteins was 3%, while 75% and 99.65% of these were quantified with <5% and <11% CoV, respectively (**Supplementary Figure S1B**). The reproducibility of this workflow was further verified by calculating the correlation of abundance values between sample pairs, yielding an *R*^2^ of 0.997, illustrating excellent technical reproducibility (**Supplementary Figure S1C**).

Pairwise comparison between (sets of) species was performed on the subset of peptides detected with high confidence in either group of samples. For instance, when comparing rodent and primate synaptosome fractions, we used the set of high quality peptides that were found in all rodent and/or all primate samples. Furthermore, we disregarded peptides that mapped ambiguously to multiple genes and peptides that were not conserved in all species of the pairwise comparison.

Protein abundances were computed by the summation of their respective peptide peak areas and Loess normalized. As a result, we confidently quantified 6021 peptides covering 1642 proteins over all samples when comparing rodents with primates, and 8243 peptides covering 2109 proteins when comparing mouse with human.

Finally, the SWATH data for biological replicates in each pairwise comparison of species was subjected to quality control in order to validate reproducibility. The coefficient of determination between pairs of biological replicates was at least 0.97 for all species in all the pairwise comparisons (**Supplementary Figure S2**). Unsupervised hierarchical clustering of the log-transformed protein abundances showed grouping of samples from the same species (**Supplementary Figure S3A**). When comparing rodents with primates, the 1642 proteins compared over all species, show a median CoV 8%, 8%, 12% and 9% for mouse, rat, marmoset and human, respectively (**Supplementary Figure S3B**). The mouse vs. human comparison yielded more proteins, i.e., 2109, because within each group the stringent quality criteria for peptides were applied to fewer samples/species. The median protein CoV in this comparison was found to be 10% for both species (**Supplementary Figure S3C**).

As an internal control for accurate measurements, we first analyzed the ratios of proteins within well-established ribosome protein complexes. Proteins residing in these functional complexes are likely evolutionary well conserved and are predicted to have fixed ratios, which should result in similar stoichiometries when comparing species. **Supplementary Figures S5A,B** shows the relative protein abundances in each sample, in which we see differences between species for ribosomal proteins. However, the vast majority of values seem to move in a similar pattern among species. And indeed, visualizing the protein-protein correlations among ribosomal proteins (**Supplementary Figures S5C,D**) indicates that the relative abundance values of proteins in the ribosomal complex are tightly coupled for the vast majority of all 48 quantified proteins from the small and large subunits of the ribosome, internally validating our quantitative approach. While the relative abundance values obtained from label-free proteomics are not accurate estimates of true copy numbers (absolute abundances), we can infer that their stoichiometry (ratios of abundances across species) is fixed if up/down shift between species is tightly correlated. The only exceptions were RPS17 (enriched in rat, unlike other subunits) and RPL14 (anti-correlated with most large subunits of the ribosome).

### Comparing Synaptic Proteomes Among Species

**FIGURE 2:**
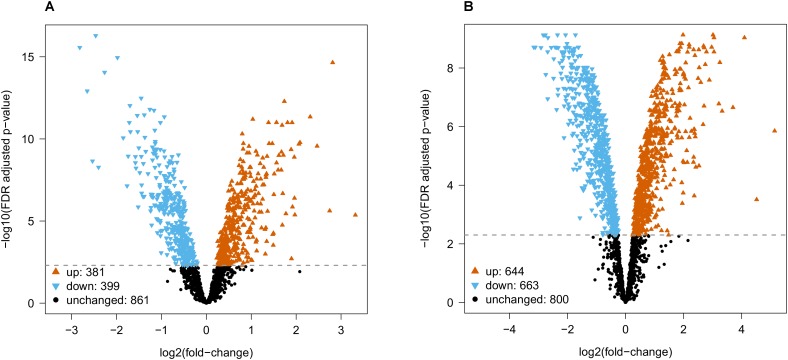
Differential abundance analysis for **(A)** rodent vs. primate and **(B)** mouse vs. human. Empirical Bayes moderated t-statistics followed by a FDR adjusted *p*-value 0.005 cutoff resulted in 399/381 proteins with decreased/ increased abundance from rodent to primate, and 663/644 proteins with decreased/increased abundance from mouse to human.

Differential abundance analysis by empirical Bayes moderated t-statistics was used to compare (sets of) species. Comparing rodents with primates resulted in 381 proteins with higher abundance in primates and 398 proteins with higher abundance in rodents, whereas 862 proteins were not significantly different at an FDR adjusted *p*-value 0.005 (**Figure 2A** and **Supplementary Table S2**). Amongst the highest differentially expressed proteins between rodents and primates belong, for instance, the cell adhesion molecules NCAM1 and -2, the sodium channel subunit SCN3B, the Annexins ANXA1 and -2 and the isocitrate dehydrogenases (IDH3A and IHD2), the latter of which are proteins with lower and higher expression in primates respectively (**Supplementary Table S2**).

When comparing mouse with human, we found 644 proteins with higher abundance in human and 663 proteins with higher abundance in mouse (in total 1307 proteins were changed), whereas 800 proteins were not differentially abundant at FDR adjusted *p*-value 0.005 (**Figure 2B** and **Supplementary Table S3**). The fold-changes for proteins that were not differentially abundant were similar across all species comparisons (**Supplementary Figure S7** and **Supplementary Tables S2**–**S4**). Proteins that were statistically significant had much higher fold-changes and varied between species comparisons (eg., differences in mouse vs. human were stronger than in mouse vs. rat).

Both statistical tests indicated a large number of proteins that were differentially abundant. In particular, the low variation between biological replicates (see quality control, (**Supplementary Figures S2**, **S3** and **Supplementary Tables S2**, **S3**) allowed us to reliably detect fold changes between species, i.e., the lowest fold change for differentially abundant proteins between rodents vs. primates and mouse vs. human was 1.148 and 1.184, respectively. Obviously, it remains to be seen whether differences that are so small are biologically meaningful. If additional filtering of statistical results by fold-change is desired, we would recommend using the data tables (**Supplementary Figure S7** and **Supplementary Tables S2**–**S4**) for further filtering on fold change.

Overrepresentation analysis of differentially abundant proteins in **Figures 2A,B** in the Gene Ontology (GO) cellular components, biological processes, molecular functions and PANTHER protein classes yielded no results. Similarly, using subsets of statistically significant hits with large quantitative differences between species (fold-change of at least 2 or 3) yielded no results. Thus, functional annotations of synaptic proteins available in public databases could not explain the many species differences we detected.

However, visualization of proteins differentially expressed in mouse vs. human using the synapse model that features 336 proteins (cf. **Figure 1**) suggested interesting expression differences for functionally and structurally related proteins (**Supplementary Figure S4**). For instance, a downregulation is apparent for synaptic vesicle endocytosis and postsynaptic density proteins while upregulated groups include neurofilaments and extracellular matrix proteins. Given that synaptic proteins of interest in this study are lacking GO annotation coverage at this time we focussed on functionally related groups of proteins that are commonly studied in the synapse field to interpret species differences. We compared protein abundance profiles within functional groups of proteins in the synapse, both in terms of expression levels and correlations thereof.

### The Presynaptic Protein Groups

#### The Synaptic Vesicle

**FIGURE 3:**
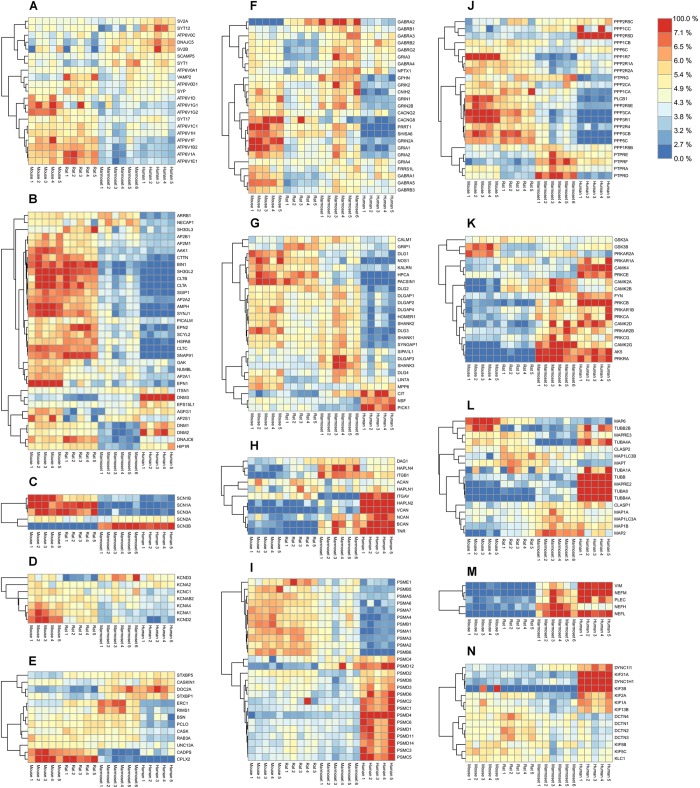
Quantified proteins in various functional groups of interest. Abundance values were scaled by their total over all samples to reveal their relative enrichment, if any, between species. The color legend on the top-right shows the protein color gradient from relatively low abundances in blue to relative enrichment in red. **(A)** Synaptic Vesicle. **(B)** Endocytosis. **(C)** Sodium channels. **(D)** Potassium channels. **(E)** Presynaptic scaffold. **(F)** Ligand-gated ion channels and associated proteins. **(G)** Postsynaptic density. **(H)** Extracellular matrix. **(I)** Proteasome. **(J)** Phosphatases. **(K)** Kinases. **(L)** Microtubules. **(M)** Neurofilaments. **(N)** Motor proteins. Respective protein-protein Pearson correlation matrices are shown in **Supplementary Figure S6**.

Synaptic transmitters are pumped into synaptic vesicles using a proton gradient generated by the vesicular-ATPase. The ATPase is built of the vesicle external V1 domain and the transmembrane V0 domain each consisting of different subunits (ATP6V1- and ATP6V0- subunits, respectively). The v-ATPase shows no differential expression of the proton translocating V0 domain subunits between species. In contrast a higher expression of the ATPase V1 domain subunits is observed in both rodent species (**Figure 3A**). When comparing the expression of ATP6V subunits in mouse and human, the V0 and V1 subunits each show tight co-expression (**Supplementary Figure S6A**). The stator subunit c, involved in assembly of subunits and regulator of the activity of the v-ATPase, shows a distinct pattern from other subunits.

When inspecting other proteins of the vesicle, three major expression-correlated subgroups can be discerned. Next to the ATP6-ase subgroup, a set of 6 integral synaptic vesicle membrane proteins, and a group containing synaptophysin (SYP) and its interacting protein synaptobrevin (VAMP2) together with two vATP6V0 subunits (**Figure 3A**) is identified. Interestingly, SYP and VAMP2 have been shown previously to interact with the vATP6V0 subunits (Galli et al., 1996). Also, these proteins correlate with the ATP6V1 group. The set of 6 integral vesicle membrane proteins are highly correlated amongst each other and anti-correlated in expression with the ATP6V1 group.

#### The Endocytosis Machinery

Core proteins of the synaptic endocytosis machinery (McMahon and Boucrot, 2011) show a differential regulation over species. Notably many endocytosis proteins were abundantly expressed in rodents compared to marmoset and were even lower expressed in human. The dynamins (DNM1-3) are part of a subgroup, of which members are highly expressed in human (**Figure 3B**). From this group, the intersectin1 (ITSN1) protein has been investigated regarding its role in membrane fusion and is involved in exocytosis or endocytosis (Gubar et al., 2013). The correlation matrix indicates an anti-correlation with the core endocytosis set (**Supplementary Figure S6B**). The same holds for the ITSN1-interacting protein EPS15L1. When inspecting the correlation of ITSN1 vs. exocytosis proteins, ITSN1 correlates highest with STXBP1 (0.72), STX1A (0.63), SYT1 (0.55), which could suggest a role in exocytosis.

#### Ion Channels

Sodium channels are formed by the major pore forming alpha subunits (SCN1-4A) and the channel modulating beta subunits (SCN1-4B). Four out of five detected subunits in hippocampus have a very strong differential expression profile between rodents and primates (**Figure 3C** and **Supplementary Figure S6C**). Also, SCN3B and SCN1B, both modulatory subunits of the sodium channels are differentially expressed in rodents and humans. Like sodium channels, K+ channels are also involved in shaping membrane depolarization. Interestingly, like the Na^+^ channels also the K^+^ channels show expression differences that relate to neuronal functional differences between rodents and primates (**Figure 3D** and **Supplementary Figure S6D**).

#### Presynaptic Organization

Organizing proteins of the vesicle release in the presynapse show differential distribution between species. Some of these such as CDPS, CPLX2, DOC2A, and STXBP1 show a differential expression between rodents and primates, e.g., with DOC2A high and CPLX2 low in primates (**Figure 3E**). Various presynaptic proteins show the lowest expression in human. A strong positively correlated expression cluster exists for the presynaptic scaffold organizers, Piccolo (PCLO), CASK, Bassoon (BSN), and RIMS1, and the RIMS1 binding protein ERC1. The group of CASKIN1, STXBP1, STXBP5, and DOC2A is anti-correlated with the expression cluster CDPS, UNC13A, complexin2 and Rab3A (**Supplementary Figure S6E**).

### The Postsynaptic Protein Groups

#### Ligand-Gated Ion Channels

When exploring the ligand gated ion channels, in particular the AMPAR, NMDAR, GABAR, and their associated proteins, striking differences in expression patterns between 4 species are observed. In particular, GRIA1, 2, and its auxiliary proteins (Schwenk et al., 2012; Chen et al., 2014) CACNG2, PRRT1, CNIH2, and SHISA6 have low expression in human (**Figure 3F**). This is different for the GABAR subunits for which expression is lowest in rat. A strong expression cluster exists for GABAR α1/β2/γ2, which is considered a typical GABA_A_ receptor subunit composition, and is distinct from GABAR α5/β3 and GABAR α2/β1. Interestingly, GABAR α5/β3 form a genomic subunit cluster (Papadimitriou et al., 2001) and have been described as part of a single functional receptor (Sur et al., 1998) (**Supplementary Figure S6F**).

#### Postsynaptic Density Proteins

A fraction of the postsynaptic density (PSD) proteins show a low abundance in humans compared to rodents and marmoset (**Figure 3G**). There are no PSD proteins that are consistently differentially expressed between rodent and primates. NSF, involved in fusion of AMPARs with the postsynaptic membrane is highest in human. The strongest co-expression cluster is formed by NSF and PICK1, which are well-known interactors and regulators of AMPAR trafficking (Hanley et al., 2002). Also, SIPA1L1, SHANK3, DLGAP3 show co-expression. In agreement with this, SHANK3, DLGAP3 have been shown to interact (Tu et al., 1999), and both proteins bind and recruit SIPA1L1 to synapses with a central coiled-coil region that harbors a leucine zipper motif (Wendholt et al., 2006). The largest correlated cluster contains HOMER1, SHANK1, -2, DLGAPs1, -2, SYNGAP1, DLG2, -3, most of which are known to interact, e.g., Shank1-Homer1 (Tu et al., 1999), SHANK1- DLGAP1 (Im et al., 2003), DLGAP1-DLG4 (Kim et al., 1997). A subgroup is formed by HPCA, NOS1, KALRN, DLG1, PACSIN1 (**Figure 3J** and **Supplementary Figure S6G**). These proteins are not known to bind each other.

### Pan-Synaptic Protein Groups

#### The Extracellular Matrix (ECM)

The ECM plays an important role in plasticity processes. Some of the proteins of the ECM are expressed at high levels in human, in particular Versican (VCAN), Hyaluronan link protein HAPLN2 and Tenascin-R, and to some extent Neurocan (NCAN) (**Figure 3H**). These 4 proteins and HAPLN1 form a co-expressed core of the ECM (**Supplementary Figure S6H**). HAPLN2 and HAPLN4 are known to bind Brevican and Neurocan, respectively (Spicer et al., 2003). Versican was shown to increase expression in humans during progression of Alzheimer’s disease (Hondius et al., 2016).

#### The 26S Proteasome

Comparing human and mouse we found that all 10 PSMA-B proteins of the 20S core complex were differentially expressed from the 15 PSMC-D proteins of the 19S regulatory complex **Figure 3I**, Pearson correlation matrix shown in **Supplementary Figure S6I**. Although the proteasome is differentially expressed between tissues and during development (Claud et al., 2014), differential expression of 19S and 20S subunits has not been observed previously.

#### Phosphatases and Kinases

Overall, phosphatases are less expressed in primates than in rodents, with the notable exception of PPP2R5d, a phosphatase 2A regulatory subunit B family, which is highly expressed in human (**Figure 3J**). Protein phosphatase 2A is one of the four major Ser/Thr phosphatases, and disruptive mutations in the regulatory subunit PPP2R5d were found causative in prenatal overgrowth and intellectual disability (Loveday et al., 2015). Membrane bound receptor-type tyrosine-protein phosphatases (PTPRs) form a strongly co-expressed group, most abundantly expressed in marmoset, which are anti-correlated with the main group of Ser/Thr phosphatases (**Supplementary Figure S6J**).

The kinases are found higher expressed in primates than in rodents (**Figure 3K**). Interesting individual expression differences exist for some kinases. PRKAR2A, the regulatory subunit of PKA, is low expressed in human, whereas its paralog PRKAR2B is highly expressed. Remarkable expression differences exist between mouse and human for CAMK2A/B, and PRKCB.

#### The Cytoskeleton

In particular, the tubulins TUBA8 and TUBB4A are highly expressed in human (**Figure 3L**) and strongly co-expressed between species (**Supplementary Figure S6M**). MAPT and MAP6 both bind to stable microtubules and they form a co-expressed pair. Both proteins are well known for affecting cognitive abilities upon changing expression. CLASP1 and 2 are microtubule end-binding proteins (Mimori-Kiyosue et al., 2005), however, they have also been shown to bind to actin and were proposed to link actin to microtubules (Tsvetkov et al., 2007). They show anti-correlated expression suggesting these modulate opposite function with regards to the cytoskeleton species (**Supplementary Figure S6M**). In contrast to the microtubular network, actin, and adhering proteins are not strongly differentially regulated between species. Elements of the filamentous cytoskeleton neurofilaments (NEFL, NEFM) and vimentin (VIM) are all more abundantly expressed in primates than in rodents (**Figure 3M** and **Supplementary Figure S6M**).

#### Motor Proteins

Molecular motors and their adaptors serve transport functions along the cytoskeleton. KIF3b, for membrane organelle transport, and Kif21a, involved in axonal transport, and DYNC1H1, dynein, are all highly expressed in human (**Figure 3N**). Microtubular kinesin motors KIF5b, 5c and their interactor protein KLC1 are strongly co-expressed (**Supplementary Figure S6N**). The same holds for the entire group of Dynactins (DCTN1-4), viewed as adaptor proteins of Dynein (Urnavicius et al., 2015), a major motor protein of the tubulin-based transport.

### Plasticity-Related proteins

Cognitive differences among species may be underlain by the differences in the protein composition of the synapses, and that the plastic changes of hippocampal synapse physiology are considered to be at the basis of learning and memory. We thus tested whether variation in synaptic protein abundance between species might specifically involve proteins that are prone to condition-dependent synaptic plasticity. In particular, we tested the hypothesis whether this set of plasticity proteins is differentially expressed between mouse and humans; following the reasoning that synaptic plasticity might relate to a set of proteins that in recent adapted its expression to improve dynamic response to changes in synaptic stimulation. We previously defined a set of 400 synaptic plasticity proteins in mouse hippocampus, which respond to a learning stimulus with changes in expression, using a quantitative iTRAQ proteomics analysis (Rao-Ruiz et al., 2015). Of these synaptic plasticity proteins in the SWATH interspecies comparison data set, 340 were quantified in the rodent with primate comparison of which 188 proteins were differentially expressed at FDR adjusted *p*-value 0.005, which is a significant difference (Chi-square *p*-value 1.3 × 10^-03^) among all differentially abundant proteins. Analogous pairwise comparison of mouse with human and rat with human also yielded a significant difference for plasticity proteins (Chi-square *p*-values 6.0 × 10^-04^ and 1.2 × 10^-03^, respectively). One might reason that plasticity proteins by nature show more variation in expression and therefore might have an increased likelihood of being differentially abundant. Therefore, we analyzed the same synaptic plasticity proteins in the SWATH interspecies comparison of mouse with rat and found a much lower significance level at Chi-square *p*-value 0.012. Finally, we tested plasticity proteins in the marmoset with human comparison, yielding a significant difference (Chi-square *p*-value 7.0 × 10^-03^). When the group of 340 synaptic plasticity genes is assessed in the group mouse, rat, marmoset vs. human (**Supplementary Table S5**), 175 proteins show differential regulation with a FDR corrected *p*-value < 0.01. In this group of proteins some interesting sets of proteins show lower expression in human, e.g., proteins involved in endocytosis (SNAP91, SYNJ1, CLTA/B/C, and Pacsin-1), the ionotropic glutamate receptors (GRIA1 and -2) and auxiliary subunits (PRRT1, Shisa6 and CACNG8). Proteins that show higher expression in human include the extracellular matrix components (TNR, BCAN, and NCAN). In conclusion, proteins that show stimulus-dependent changes in expression after fear-learning in the mouse hippocampus are showing a differential abundance between rodents and primates and between marmoset and human, whereas comparison within rodent species does not show this.

## Discussion

In this study we used SWATH to quantify levels of hippocampal synaptic proteins of four species, the rodents; mouse and rat, and the primates; marmoset and human. We revealed many protein abundance differences between species with in many instances small fold changes. The quantification accuracy of SWATH and low technical variability of the method, allowed us to very accurately quantify even minor (as low as 1.15-fold) differences across species. Furthermore, our SWATH proteomics required building spectral libraries, which serve as catalogs for synaptic proteins in these four species analyzed. One can use these catalogs to generate protein maps of synapses to gain insight in stoichiometries (cf. **Figure 1**) or visualize differences in levels between species (cf. **Supplementary Figure S4**).

*In silico* approximations of absolute abundances from label-free data, such as iBAQ (Schwanhausser et al., 2011) or SCAMPI (Gerster et al., 2014) are currently not sufficiently accurate to detect small differences in protein abundance expected between species. As distinct peptide sequences have different mass spectrometric properties, such as ionization efficiency, their ion intensity measured by label-free proteomics can differ up to several orders of magnitude even if the absolute amount of these peptides in the input sample is the same. Including peptide standards in the sample to obtain accurate estimates of absolute abundances (Gerber et al., 2003) is not feasible for thousands of unique peptides present in the synaptic proteome of multiple species. However, physical properties may vary amongst different peptides; identical peptides from different samples behave the same. Therefore, in this study, we considered the set of peptide sequences that are identical in all species (within a given comparison of species) and quantified these confidently in all samples. This allowed comparison of the relative abundance of peptides and proteins with the highest accuracy possible. In the current study we compared synaptic fractions obtained from different species. This may involve different efficiencies of isolating the synaptosome fraction, or proteins therein, which cannot be easily corrected for. We did not observe apparent species differences in the amount of protein isolated in the synaptosome fractions. Another aspect of this study is that it inherently incorporates different postmortem delay times, in particular between human vs. marmoset and rodent brains. We cannot rule out that this may have introduced differences in the synaptic proteins isolated.

Key to the human brain’s unique capacities is probably is not its absolute or relative size, or even its number of neuronal and glia cells. Instead, this likely involves evolved neural cell types, and expanded and/or more complex patterns of neuronal connectivity. However, these specific evolutionary adaptations likely depend on the increased diversity of molecular expression signatures, both in terms of levels and of cell type specific expression. Previous transcriptome analyses have reported that there are more genes higher expressed in the adult human brain than in the non-human primate brain, and not in other examined tissues (Caceres et al., 2003; Gu and Gu, 2003; Khaitovich et al., 2004). Also it was found that there is little evidence for accelerated divergence in gene expression in the human brain (Hsieh et al., 2003). Other studies have reported several interesting findings on human-specific differences in the expression of genes involved in metabolism (Uddin et al., 2008; Babbitt et al., 2010) and on genes that are organized into human-specific co-expressed modules (Oldham et al., 2006; Konopka et al., 2012). Human-specific differences in gene expression have also been reported for groups of genes with developmental time-shifts (Somel et al., 2009), miRNA regulation (Somel et al., 2009; Hu et al., 2011), RNA editing (Li et al., 2013), and transcription factor regulation (Liu et al., 2012). Notwithstanding the usefulness of gene expression analysis, a constraint in unambiguous translation of transcript levels to proteins is the multitude of regulatory steps in protein synthesis and breakdown. Furthermore, differences in gene expression are difficult to translate to synaptic protein expression. Typically, synaptic proteins, as studied here, are well known to be regulated by condition-dependent trafficking, local synthesis and target specific breakdown.

One of the outstanding questions regarding species comparison is whether expression differences of distinct proteins or protein groups might be related to functional differences between rodents and primates. We indeed detected a number of synaptic functional groups that show differential abundance in rodents and primates. An interesting group of proteins to consider are the sodium channels, which are formed by the major pore forming alpha subunits (SCN1-4A) and the channel modulating beta subunits (SCN1-4B). Four out of five subunits detected in hippocampus have a very strong differential expression profile between rodents and primates (cf. **Figure 3C**). As such these may qualify for distinct physiology of human (mammalian) neurons. For instance, we found SCN3B and SCN1B, both modulatory subunits of the sodium channels highly differentially expressed in rodents and humans (cf. **Figure 3C**). Given the essential role of these channels in the formation and propagation of action potentials, this might be an important observation. Co-expression studies of SCN1B, -2B, and -3B subunits with the SCN2A subunit in HEK293 cells have shown to shift sodium channel activation and inactivation to more positive membrane potentials (Qu et al., 2001). However, SCN3b is unique in causing increased persistent sodium currents. Because persistent sodium currents are thought to amplify summation of synaptic inputs, expression of this subunit would increase the excitability of the expressing neurons to all of their inputs. This might be specifically the case for human neurons. Interestingly, SCN2A and SCN3B are highly co-expressed over the species, suggestive of a co-expressed pair (cf. **Supplementary Figure S6C**). If so, the not differentially expressed SCN2A channel might be modulated by SCN3B thereby providing a higher offset for neuronal excitation in humans.

Among proteins with differential level we found neurofilament, which is particularly highly expressed in human brain tissue. Synaptosome fractions are only enriched in synaptic proteins and therefore caution is warranted regarding inferring neurofilament presence at the synapse based on biochemical means only. Neurofilament has been regarded as an impurity of the synaptic fraction [e.g., (Matus et al., 1980)], however, recent immuno-EM data shows its presence at striatal postsynaptic sites (Yuan et al., 2015). We therefore consider it a potential component of the hippocampal synapse from which it can be readily isolated [this study, (Yuan et al., 2015)]. Deletion of neurofilaments has been shown to affect synaptic long-term potentiation (Yuan et al., 2015) and neurofilament expressing pyramidal neurons in humans may show higher vulnerability to neurodegenerative disease (Perrot et al., 2008).

Importantly, we demonstrated that, among the many proteins with expression differences, a group of plasticity–related proteins follows a global up-regulation in human hippocampal synapse proteome. One might argue that an important aspect of species evolution is the potential to be adaptive, at the molecular and subcellular level displayed as synaptic plasticity. Plasticity mechanisms enable the brain with the feature to rapidly change the efficacy of synapses, allowing these to be used over a wide dynamic range of signal transmission and act as logical operators. In line with this, synaptic plasticity might relate to a set of proteins that evolved rapidly in recent evolution. Changes in protein abundance might reflect dynamic response to changes in synaptic stimulation, and/ or change the impact a protein has in a larger network. As such this set is expected to be dynamic within a species and consequently may show abundance differences between species. To specifically test this we used a plasticity set of proteins as found regulated by a strong fear learning paradigm impacting on the hippocampus (Rao-Ruiz et al., 2015). Using this set of proteins we asked whether its constituents would belong to the rodent-primate conserved or rather the differentially expressed part of the synaptic proteome. The latter was true. This indicates that within the synaptic proteome those proteins of which expression differences maximally evolved during evolution are overrepresented in the plasticity response.

## Data Availability Statement

The datasets generated for this study can have been deposited to the ProteomeXchange Consortium via the PRIDE (Vizcaino et al., 2016) partner repository with the dataset identifier PXD009251.

## Ethics Statement

The use of rodent brain material in this study was carried out in accordance with the recommendations of the animal ethics committee of the Vrije Universiteit Amsterdam. The protocol was approved by the animal ethics committee of the Vrije Universiteit Amsterdam. The use of marmoset brain material in this study was carried out in accordance with the recommendations of the Biomedical Primate Research Centre (BPRC) ethics committee before the start of experiments. The protocol was approved by the Biomedical Primate Research Centre (BPRC) ethics committee before the start of experiments, according to Dutch law. Human hippocampus brain samples with donor consent were obtained from, and used according to the guidelines of, the Dutch Brain Bank. All Dutch Brain Bank procedures have been approved by the ethics committee of the VU University Medical Center (Amsterdam, Netherlands). This study was carried out in accordance with the recommendations by the ethics committee of VU University Medical Center. The protocol was approved by the ethics committee of VU University Medical Center. All subjects gave written informed consent in accordance with the Declaration of Helsinki.

## Author Contributions

FK, NP, KL, and AS planned and designed the experiments. NP and IrP performed the experiments. FK performed the data analysis. SF and InP provided the marmoset samples. FK, KL, NP, and AS wrote the manuscript. All authors reviewed the manuscript.

## Conflict of Interest Statement

The authors declare that the research was conducted in the absence of any commercial or financial relationships that could be construed as a potential conflict of interest.

## Supplementary Material

FIGURE S1 Reproducibility of three technical replicate SWATH measurements of mouse synaptosomes. **(A)** Peptide Coefficient of Variation. **(B)** Protein Coefficient of Variation. **(C)** pair-wise scatterplots for log10 protein abundance values, with abundance distribution of each sample shown on the diagonal.

FIGURE S2 Reproducibility of biological replicate SWATH measurements for **(A)** mouse, **(B)** rat, **(C)** marmoset, and **(D)** human for the set of proteins used in the rodent vs. primate comparison in **Figure 1A**. Pair-wise scatterplots for log10 protein abundance values, with abundance distribution of each sample shown on the diagonal.

FIGURE S3 **(A)** Hierarchical clustering of the Euclidean distance between protein abundances in the rodent vs. primate comparison using Ward’s D2 method. **(B)** Protein Coefficient of Variation for all proteins used in the rodent vs. primate comparison in **Figure 1A** and textbf(C) for the mouse vs. human comparison in **Figure 1B**.

FIGURE S4 Differentially abundant proteins between mouse and human synaptosomes highlighted in a model of the synaptic proteome (c.f. **Figure 1**). In total 1307 proteins were changed between these species at FDR adjusted *p*-value 0.005 (**Figure 2B** and **Supplementary Table S3**), which corresponded to 234 out of 336 proteins in this model. Protein colors reflect the fold changes from mouse to human (e.g., the first light blue color in the scale bar depicts proteins 1.18 to 1.55-fold more abundant in mouse).

FIGURE S5 Quantified proteins in ribosomal protein complexes (top row) and the respective protein-protein Pearson correlation matrices (bottom row). Abundance values were scaled by their total over all samples to reveal their relative enrichment, if any, between species. The Pearson correlations were computed for pairs of proteins using their abundance values from all samples. Strong correlations are shown in dark purple while anti-correlations are shown in green, as visualized in the legend on the bottom-right. **(A,C)** small subunits of ribosomal proteins. **(B,D)** large subunits of ribosomal proteins.

FIGURE S6 Correlation matrices for protein groups shown in **Figure 3**. The Pearson correlations were computed for pairs of proteins using their abundance values from all samples. Strong correlations are shown in dark purple while anti-correlations are shown in green, as visualized in the legend on the top-right. **(A)** Synaptic Vesicle. **(B)** Endocytosis. **(C)** Sodium channels. **(D)** Potassium channels. **(E)** Presynaptic scaffold. **(F)** Ligand-gated ion channels and associated proteins. **(G)** Postsynaptic density. **(H)** Extracellular matrix. **(I)** Proteasome. **(J)** Phosphatases. **(K)** Kinases. **(L)** Microtubules. **(M)** Neurofilaments. **(N)** Motor proteins.

FIGURE S7 Protein abundance fold-changes for various species comparisons. The subset of differentially abundant proteins (at FDR adjusted *p*-value 0.005) is shown in orange while remaining proteins are shown in white. The median fold-change over unchanged proteins from all comparisons is 1.125 (0.17 on log2 scale), as visualized by a horizontal dashed line.

TABLE S1 Protein iBAQ abundance values for discovery proteomics (DDA) measurements of mouse, rat, marmoset and human synaptosomes used for the spectral library.

TABLE S2 SWATH data tables for the rodent vs. primate comparison in **Figure 2A**.

TABLE S3 SWATH data tables for the mouse vs. human comparison in **Figure 2B**.

TABLE S4 SWATH data tables for the mouse vs. rat comparison in **Supplementary Figure S7**.

TABLE S5 SWATH data tables for the mouse, rat, marmoset vs. human comparison in “Plasticity-Related Proteins.”

## References

[B1] Babbitt C. C. Fedrigo O. Pfefferle A. D. Boyle A. P. Horvath J. E. Furey T. S. (2010). Both noncoding and protein-coding RNAs contribute to gene expression evolution in the primate brain. *Genome Biol. Evol.* 2 67–79. 10.1093/gbe/evq002 20333225 PMC2839352

[B2] Bayes A. Collins M. O. Reig-Viader R. Gou G. Goulding D. Izquierdo A. (2017). Evolution of complexity in the zebrafish synapse proteome. *Nat. Commun.* 8:14613. 10.1038/ncomms14613 28252024 PMC5337974

[B3] Bruderer R. Bernhardt O. M. Gandhi T. Miladinovic S. M. Cheng L. Y. Messner S. (2015). Extending the limits of quantitative proteome profiling with data-independent acquisition and application to acetaminophen-treated three-dimensional liver microtissues. *Mol. Cell. Proteomics* 14 1400–1410. 10.1074/mcp.M114.044305 25724911 PMC4424408

[B4] Caceres M. Lachuer J. Zapala M. A. Redmond J. C. Kudo L. Geschwind D. H. (2003). Elevated gene expression levels distinguish human from non-human primate brains. *Proc. Natl. Acad. Sci. U.S.A.* 100 13030–13035. 10.1073/pnas.2135499100 14557539 PMC240739

[B5] Chen N. Pandya N. J. Koopmans F. Castelo-Szekelv V. van der Schors R. C. Smit A. B. (2014). Interaction proteomics reveals brain region-specific AMPA receptor complexes. *J. Proteome Res.* 13 5695–5706. 10.1021/pr500697b 25337787

[B6] Chua J. J. (2014). Macromolecular complexes at active zones: integrated nano-machineries for neurotransmitter release. *Cell. Mol. Life Sci.* 71 3903–3916. 10.1007/s00018-014-1657-5 24912984 PMC11113288

[B7] Chua J. J. Kindler S. Boyken J. Jahn R. (2010). The architecture of an excitatory synapse. *J. Cell Sci.* 123(Pt 6), 819–823. 10.1242/jcs.052696 20200227

[B8] Claud E. C. McDonald J. A. He S. M. Yu Y. Duong L. Sun J. (2014). Differential expression of 26S proteasome subunits and functional activity during neonatal development. *Biomolecules* 4 812–826. 10.3390/biom4030812 25177858 PMC4192673

[B9] Cooper D. G. (2003). *Hippocampus.* Princeton, NJ: ELS.

[B10] Cox J. Mann M. (2008). MaxQuant enables high peptide identification rates, individualized p.p.b.-range mass accuracies and proteome-wide protein quantification. *Nat. Biotechnol.* 26 1367–1372. 10.1038/nbt.1511 19029910

[B11] Galli T. McPherson P. S. De Camilli P. (1996). The V0 sector of the V-ATPase, synaptobrevin, and synaptophysin are associated on synaptic vesicles in a Triton X-100-resistant, freeze-thawing sensitive, complex. *J. Biol. Chem.* 271 2193–2198. 10.1074/jbc.271.4.2193 8567678

[B12] Gerber S. A. Rush J. Stemman O. Kirschner M. W. Gygi S. P. (2003). Absolute quantification of proteins and phosphoproteins from cell lysates by tandem MS. *Proc. Natl. Acad. Sci. U.S.A.* 100 6940–6945. 10.1073/pnas.0832254100 12771378 PMC165809

[B13] Gerster S. Kwon T. Ludwig C. Matondo M. Vogel C. Marcotte E. M. (2014). Statistical approach to protein quantification. *Mol. Cell. Proteomics* 13 666–677. 10.1074/mcp.M112.025445 24255132 PMC3916661

[B14] Gillet L. C. Navarro P. Tate S. Rost H. Selevsek N. Reiter L. (2012). Targeted data extraction of the MS/MS spectra generated by data-independent acquisition: a new concept for consistent and accurate proteome analysis. *Mol. Cell. Proteomics* 11:O111016717. 10.1074/mcp.O111.016717 22261725 PMC3433915

[B15] Gonzalez-Lozano M. A. Klemmer P. Gebuis T. Hassan C. van Nierop P. van Kesteren R. E. (2016). Dynamics of the mouse brain cortical synaptic proteome during postnatal brain development. *Sci. Rep.* 6:35456. 10.1038/srep35456 27748445 PMC5066275

[B16] Gu J. Gu X. (2003). Induced gene expression in human brain after the split from chimpanzee. *Trends Genet.* 19 63–65. 10.1016/S0168-9525(02)00040-9 12547510

[B17] Gubar O. Morderer D. Tsyba L. Croise P. Houy S. Ory S. (2013). Intersectin: the crossroad between vesicle exocytosis and endocytosis. *Front. Endocrinol.* 4:109. 10.3389/fendo.2013.00109 23986746 PMC3753573

[B18] Hanley J. G. Khatri L. Hanson P. I. Ziff E. B. (2002). NSF ATPase and alpha-/beta-SNAPs disassemble the AMPA receptor-PICK1 complex. *Neuron* 34 53–67. 10.1016/S0896-6273(02)00638-4 11931741

[B19] Hondius D. C. van Nierop P. Li K. W. Hoozemans J. J. van der Schors R. C. van Haastert E. S. (2016). Profiling the human hippocampal proteome at all pathologic stages of Alzheimer’s disease. *Alzheimers Dement.* 12 654–668. 10.1016/j.jalz.2015.11.002 26772638

[B20] Hsieh W. P. Chu T. M. Wolfinger R. D. Gibson G. (2003). Mixed-model reanalysis of primate data suggests tissue and species biases in oligonucleotide-based gene expression profiles. *Genetics* 165 747–757. 14573485 10.1093/genetics/165.2.747 PMC1462792

[B21] Hu H. Y. Guo S. Xi J. Yan Z. Fu N. Zhang X. (2011). MicroRNA expression and regulation in human, chimpanzee, and macaque brains. *PLoS Genet.* 7:e1002327. 10.1371/journal.pgen.1002327 22022286 PMC3192836

[B22] Im Y. J. Lee J. H. Park S. H. Park S. J. Rho S. H. Kang G. B. (2003). Crystal structure of the Shank PDZ-ligand complex reveals a class I PDZ interaction and a novel PDZ-PDZ dimerization. *J. Biol. Chem.* 278 48099–48104. 10.1074/jbc.M306919200 12954649

[B23] Jahn R. Fasshauer D. (2012). Molecular machines governing exocytosis of synaptic vesicles. *Nature* 490 201–207. 10.1038/nature11320 23060190 PMC4461657

[B24] Khaitovich P. Muetzel B. She X. Lachmann M. Hellmann I. Dietzsch J. (2004). Regional patterns of gene expression in human and chimpanzee brains. *Genome Res.* 14 1462–1473. 10.1101/gr.2538704 15289471 PMC509255

[B25] Kim E. Naisbitt S. Hsueh Y. P. Rao A. Rothschild A. Craig A. M. (1997). GKAP, a novel synaptic protein that interacts with the guanylate kinase-like domain of the PSD-95/SAP90 family of channel clustering molecules. *J. Cell Biol.* 136 669–678. 10.1083/jcb.136.3.669 9024696 PMC2134290

[B26] Konopka G. Friedrich T. Davis-Turak J. Winden K. Oldham M. C. Gao F. (2012). Human-specific transcriptional networks in the brain. *Neuron* 75 601–617. 10.1016/j.neuron.2012.05.034 22920253 PMC3645834

[B27] Koopmans F. Ho J. T. C. Smit A. B. Li K. W. (2018). Comparative analyses of data independent acquisition mass spectrometric approaches: DIA, WiSIM-DIA, and untargeted DIA. *Proteomics* 18:1700304. 10.1002/pmic.201700304 29134766 PMC5817406

[B28] Li Z. Bammann H. Li M. Liang H. Yan Z. Phoebe Chen Y. P. (2013). Evolutionary and ontogenetic changes in RNA editing in human, chimpanzee, and macaque brains. *RNA* 19 1693–1702. 10.1261/rna.039206.113 24152549 PMC3884655

[B29] Liu X. Somel M. Tang L. Yan Z. Jiang X. Guo S. (2012). Extension of cortical synaptic development distinguishes humans from chimpanzees and macaques. *Genome Res.* 22 611–622. 10.1101/gr.127324.111 22300767 PMC3317144

[B30] Loveday C. Tatton-Brown K. Clarke M. Westwood I. Renwick A. Ramsay E. (2015). Mutations in the PP2A regulatory subunit B family genes PPP2R5B, PPP2R5C and PPP2R5D cause human overgrowth. *Hum. Mol. Genet.* 24 4775–4779. 10.1093/hmg/ddv182 25972378 PMC4527483

[B31] Markov N. T. Ercsey-Ravasz M. Van Essen D. C. Knoblauch K. Toroczkai Z. Kennedy H. (2013). Cortical high-density counterstream architectures. *Science* 342:1238406. 10.1126/science.1238406 24179228 PMC3905047

[B32] Matus A. Pehling G. Ackermann M. Maeder J. (1980). Brain postsynaptic densities: the relationship to glial and neuronal filaments. *J. Cell Biol.* 87(2 Pt 1), 346–359. 10.1083/jcb.87.2.346 7000794 PMC2110744

[B33] McMahon H. T. Boucrot E. (2011). Molecular mechanism and physiological functions of clathrin-mediated endocytosis. *Nat. Rev. Mol. Cell Biol.* 12 517–533. 10.1038/nrm3151 21779028

[B34] Mesulam M. (2000). Brain, mind, and the evolution of connectivity. *Brain Cogn.* 42 4–6. 10.1006/brcg.1999.1145 10739582

[B35] Mi H. Huang X. Muruganujan A. Tang H. Mills C. Kang D. (2017). PANTHER version 11: expanded annotation data from gene ontology and reactome pathways, and data analysis tool enhancements. *Nucleic Acids Res.* 45 D183–D189. 10.1093/nar/gkw1138 27899595 PMC5210595

[B36] Mimori-Kiyosue Y. Grigoriev I. Lansbergen G. Sasaki H. Matsui C. Severin F. (2005). CLASP1 and CLASP2 bind to EB1 and regulate microtubule plus-end dynamics at the cell cortex. *J. Cell Biol.* 168 141–153. 10.1083/jcb.200405094 15631994 PMC2171674

[B37] Muller C. S. Haupt A. Bildl W. Schindler J. Knaus H. G. Meissner M. (2010). Quantitative proteomics of the Cav2 channel nano-environments in the mammalian brain. *Proc. Natl. Acad. Sci. U.S.A.* 107 14950–14957. 10.1073/pnas.1005940107 20668236 PMC2930569

[B38] Muntane G. Horvath J. E. Hof P. R. Ely J. J. Hopkins W. D. Raghanti M. A. (2015). Analysis of synaptic gene expression in the neocortex of primates reveals evolutionary changes in glutamatergic neurotransmission. *Cereb. Cortex* 25 1596–1607. 10.1093/cercor/bht354 24408959 PMC4428301

[B39] Oldham M. C. Horvath S. Geschwind D. H. (2006). Conservation and evolution of gene coexpression networks in human and chimpanzee brains. *Proc. Natl. Acad. Sci. U.S.A.* 103 17973–17978. 10.1073/pnas.0605938103 17101986 PMC1693857

[B40] Pandya N. J. Koopmans F. Slotman J. A. Paliukhovich I. Houtsmuller A. B. Smit A. B. (2017). Correlation profiling of brain sub-cellular proteomes reveals co-assembly of synaptic proteins and subcellular distribution. *Sci. Rep.* 7:12107. 10.1038/s41598-017-11690-3 28935861 PMC5608747

[B41] Papadimitriou G. N. Dikeos D. G. Karadima G. Avramopoulos D. Daskalopoulou E. G. Stefanis C. N. (2001). GABA-A receptor beta3 and alpha5 subunit gene cluster on chromosome 15q11-q13 and bipolar disorder: a genetic association study. *Am. J. Med. Genet.* 105 317–320. 10.1002/ajmg.1354 11378843

[B42] Perrot R. Berges R. Bocquet A. Eyer J. (2008). Review of the multiple aspects of neurofilament functions, and their possible contribution to neurodegeneration. *Mol. Neurobiol.* 38 27–65. 10.1007/s12035-008-8033-0 18649148

[B43] Qu Y. Curtis R. Lawson D. Gilbride K. Ge P. DiStefano P. S. (2001). Differential modulation of sodium channel gating and persistent sodium currents by the beta1 beta2 and beta3 subunits. *Mol. Cell. Neurosci.* 18 570–580. 10.1006/mcne.2001.1039 11922146

[B44] R Core Team (2014). *R: A Language and Environment for Statistical Computing.* Vienna: R Foundation for Statistical Computing.

[B45] Rao-Ruiz P. Carney K. E. Pandya N. van der Loo R. J. Verheijen M. H. van Nierop P. (2015). Time-dependent changes in the mouse hippocampal synaptic membrane proteome after contextual fear conditioning. *Hippocampus* 25 1250–1261. 10.1002/hipo.22432 25708624

[B46] Schwanhausser B. Busse D. Li N. Dittmar G. Schuchhardt J. Wolf J. (2011). Global quantification of mammalian gene expression control. *Nature* 473 337–342. 10.1038/nature10098 21593866

[B47] Schwenk J. Harmel N. Brechet A. Zolles G. Berkefeld H. Muller C. S. (2012). High-resolution proteomics unravel architecture and molecular diversity of native AMPA receptor complexes. *Neuron* 74 621–633. 10.1016/j.neuron.2012.03.034 22632720

[B48] Silbereis J. C. Pochareddy S. Zhu Y. Li M. Sestan N. (2016). The cellular and molecular landscapes of the developing human central nervous system. *Neuron* 89 248–268. 10.1016/j.neuron.2015.12.008 26796689 PMC4959909

[B49] Smyth G. K. Michaud J. Scott H. S. (2005). Use of within-array replicate spots for assessing differential expression in microarray experiments. *Bioinformatics* 21 2067–2075. 10.1093/bioinformatics/bti270 15657102

[B50] Somel M. Franz H. Yan Z. Lorenc A. Guo S. Giger T. (2009). Transcriptional neoteny in the human brain. *Proc. Natl. Acad. Sci. U.S.A.* 106 5743–5748. 10.1073/pnas.0900544106 19307592 PMC2659716

[B51] Spicer A. P. Joo A. Bowling R. A. Jr. (2003). A hyaluronan binding link protein gene family whose members are physically linked adjacent to chondroitin sulfate proteoglycan core protein genes: the missing links. *J. Biol. Chem.* 278 21083–21091. 10.1074/jbc.M213100200 12663660

[B52] Spijker S. (2011). “Dissection of rodent brain regions,” in *Neuroproteomics*, ed. Li K. W. (Totowa, NJ: Humana Press).

[B53] Sur C. Quirk K. Dewar D. Atack J. McKernan R. (1998). Rat and human hippocampal alpha5 subunit-containing gamma-aminobutyric acidA receptors have alpha5 beta3 gamma2 pharmacological characteristics. *Mol. Pharmacol.* 54 928–933. 10.1124/mol.54.5.928 9804628

[B54] Testa-Silva G. Verhoog M. B. Goriounova N. A. Loebel A. Hjorth J. Baayen J. C. (2010). Human synapses show a wide temporal window for spike-timing-dependent plasticity. *Front. Synaptic Neurosci.* 2:12 10.3389/fnsyn.2010.00012 PMC3059666 21423498

[B55] Testa-Silva G. Verhoog M. B. Linaro D. de Kock C. P. Baayen J. C. Meredith R. M. (2014). High bandwidth synaptic communication and frequency tracking in human neocortex. *PLoS Biol.* 12:e1002007. 10.1371/journal.pbio.1002007 25422947 PMC4244038

[B56] Tsvetkov A. S. Samsonov A. Akhmanova A. Galjart N. Popov S. V. (2007). Microtubule-binding proteins CLASP1 and CLASP2 interact with actin filaments. *Cell Motil. Cytoskeleton* 64 519–530. 10.1002/cm.20201 17342765

[B57] Tu J. C. Xiao B. Naisbitt S. Yuan J. P. Petralia R. S. Brakeman P. (1999). Coupling of mGluR/Homer and PSD-95 complexes by the Shank family of postsynaptic density proteins. *Neuron* 23 583–592. 10.1016/S0896-6273(00)80810-7 10433269

[B58] Uddin M. Goodman M. Erez O. Romero R. Liu G. Islam M. (2008). Distinct genomic signatures of adaptation in pre- and postnatal environments during human evolution. *Proc. Natl. Acad. Sci. U.S.A.* 105 3215–3220. 10.1073/pnas.0712400105 18305157 PMC2265188

[B59] Urnavicius L. Zhang K. Diamant A. G. Motz C. Schlager M. A. Yu M. (2015). The structure of the dynactin complex and its interaction with dynein. *Science* 347 1441–1446. 10.1126/science.aaa4080 25814576 PMC4413427

[B60] van den Heuvel M. P. Bullmore E. T. Sporns O. (2016). Comparative connectomics. *Trends Cogn. Sci.* 20 345–361. 10.1016/j.tics.2016.03.001 27026480

[B61] Vizcaino J. A. Csordas A. del-Toro N. Dianes J. A. Griss J. Lavidas I. (2016). 2016 update of the PRIDE database and its related tools. *Nucleic Acids Res.* 44 D447–D456. 10.1093/nar/gkv1145 26527722 PMC4702828

[B62] Weingarten J. Lassek M. Mueller B. F. Rohmer M. Lunger I. Baeumlisberger D. (2014). The proteome of the presynaptic active zone from mouse brain. *Mol. Cell. Neurosci.* 59 106–118. 10.1016/j.mcn.2014.02.003 24534009

[B63] Wendholt D. Spilker C. Schmitt A. Dolnik A. Smalla K. H. Proepper C. (2006). ProSAP-interacting protein 1 (ProSAPiP1), a novel protein of the postsynaptic density that links the spine-associated rap-gap (SPAR) to the scaffolding protein proSAP2/Shank3. *J. Biol. Chem.* 281 13805–13816. 10.1074/jbc.M601101200 16522626

[B64] Wilhelm B. G. Mandad S. Truckenbrodt S. Krohnert K. Schafer C. Rammner B. (2014). Composition of isolated synaptic boutons reveals the amounts of vesicle trafficking proteins. *Science* 344 1023–1028. 10.1126/science.1252884 24876496

[B65] Wisniewski J. R. Zougman A. Nagaraj N. Mann M. (2009). Universal sample preparation method for proteome analysis. *Nat. Methods* 6 359–362. 10.1038/nmeth.1322 19377485

[B66] Yuan A. Sershen Veeranna H. Basavarajappa B. S. Kumar A. Hashim A. (2015). Neurofilament subunits are integral components of synapses and modulate neurotransmission and behavior in vivo. *Mol. Psychiatry* 20 986–994. 10.1038/mp.2015.45 25869803 PMC4514553

