# Supplementary figures and images for: Comparative Hippocampal Synaptic Proteomes of Rodents and Primates: Differences in Neuroplasticity-Related Proteins
Source: Front Mol Neurosci. 2018 Oct 2;11:364. doi: 10.3389/fnmol.2018.00364 (PMC6176546; doi:10.3389/fnmol.2018.00364)

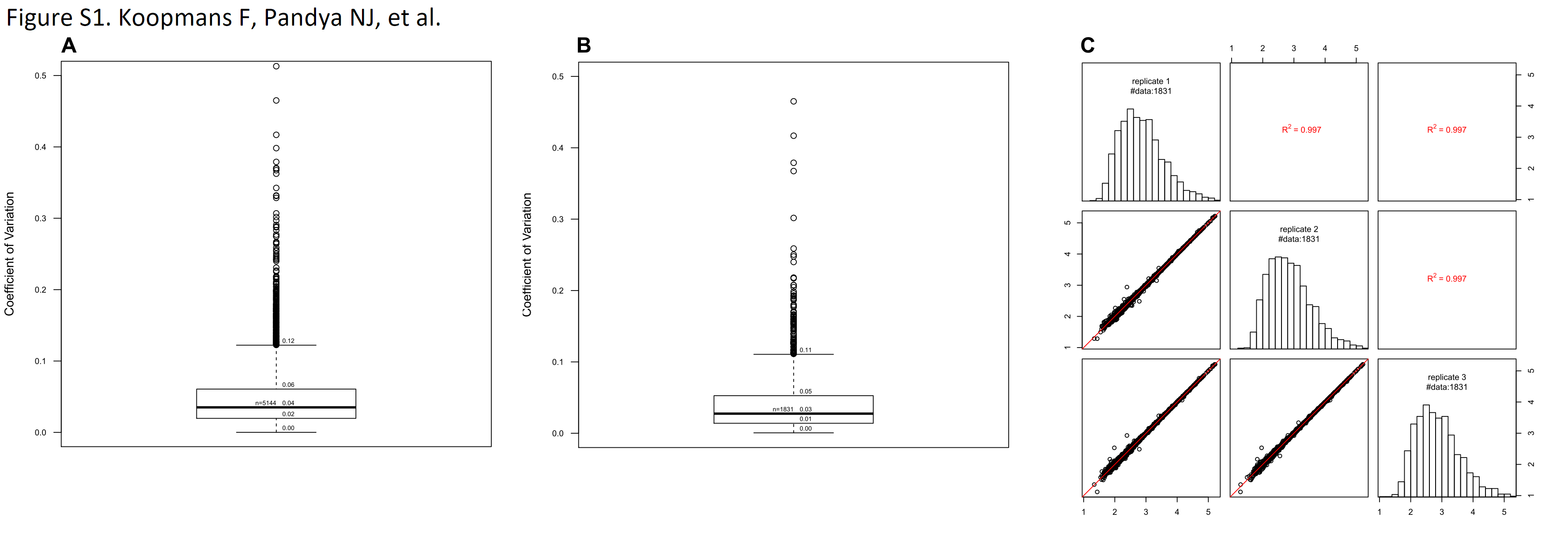

Supplement: FIGURE S1 — Reproducibility of three technical replicate SWATH measurements of mouse synaptosomes. (A) Peptide Coefficient of Variation. (B) Protein Coefficient of Variation. (C) pair-wise scatterplots for log10 protein abundance values, with abundance distribution of each sample shown on the diagonal. [file Image_1.TIFF]

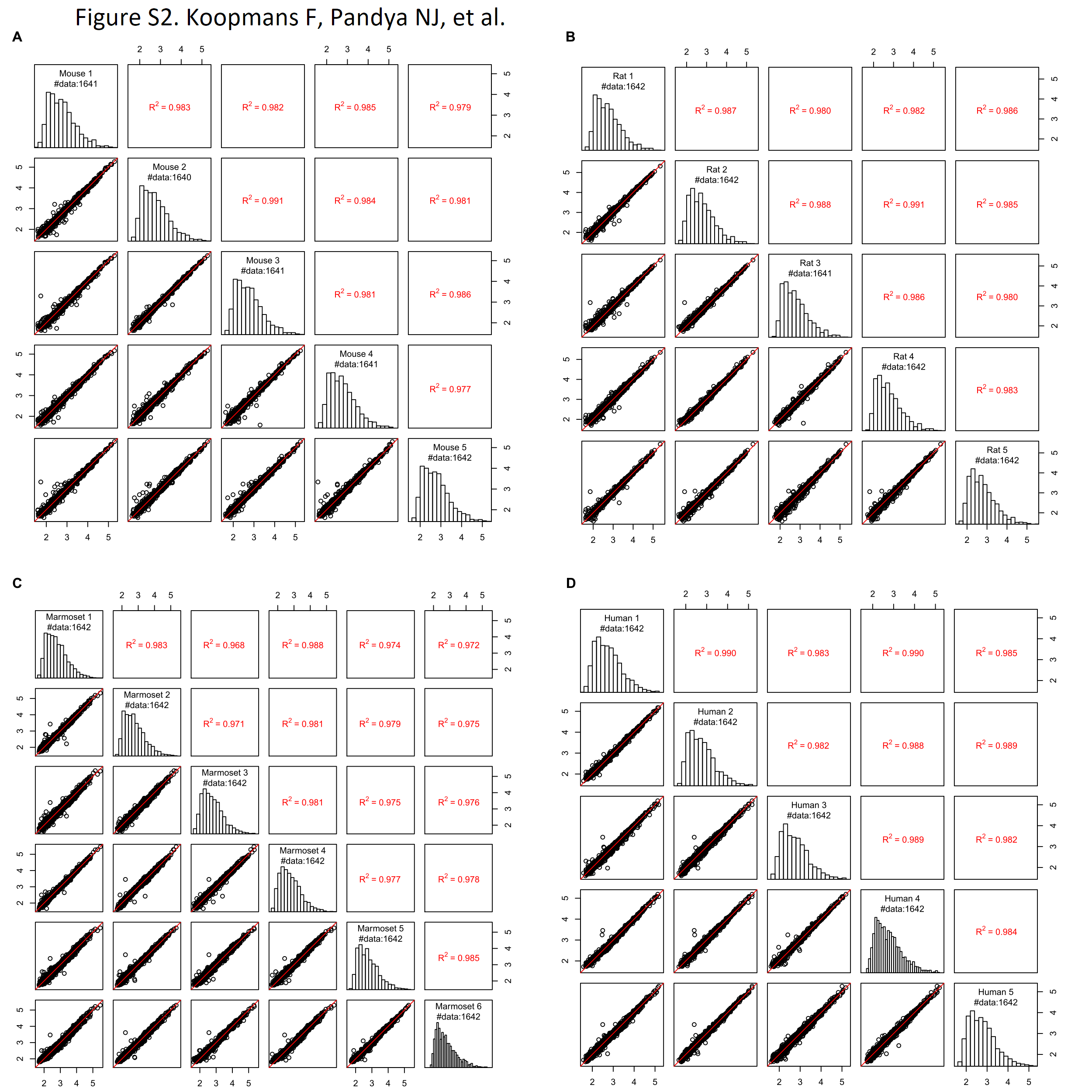

Supplement: FIGURE S2 — Reproducibility of biological replicate SWATH measurements for (A) mouse, (B) rat, (C) marmoset, and (D) human for the set of proteins used in the rodent vs. primate comparison in Figure 1A. Pair-wise scatterplots for log10 protein abundance values, with abundance distribution of each sample shown on the diagonal. [file Image_2.TIFF]

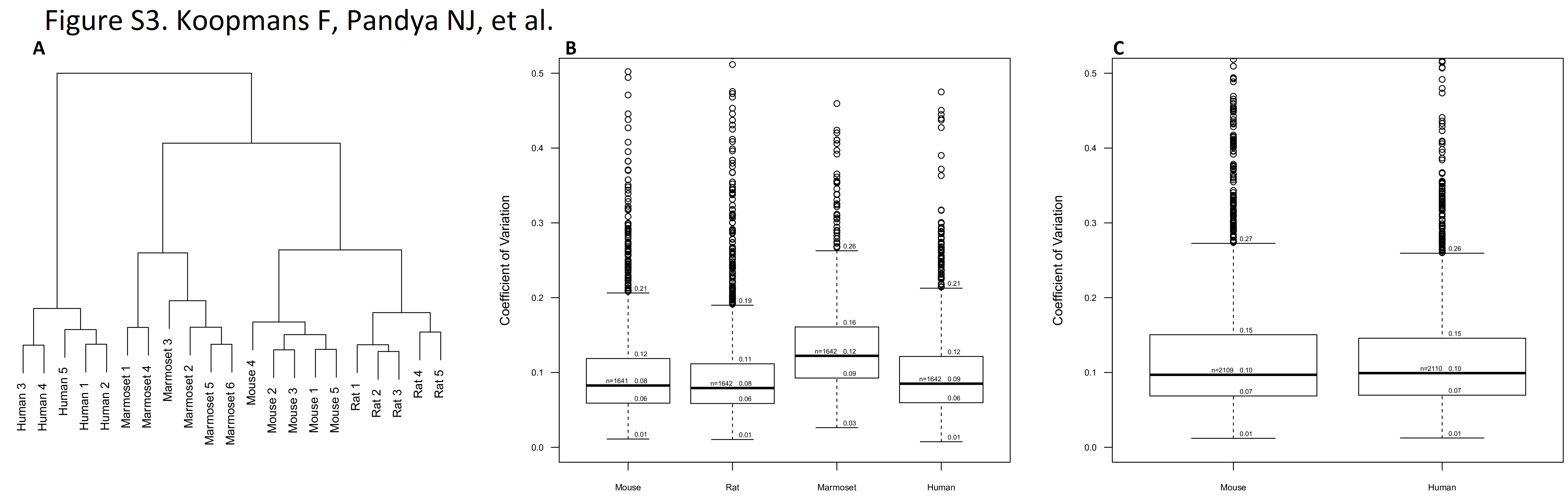

Supplement: FIGURE S3 — (A) Hierarchical clustering of the Euclidean distance between protein abundances in the rodent vs. primate comparison using Ward’s D2 method. (B) Protein Coefficient of Variation for all proteins used in the rodent vs. primate comparison in Figure 1A and textbf(C) for the mouse vs. human comparison in Figure 1B. [file Image_3.TIFF]

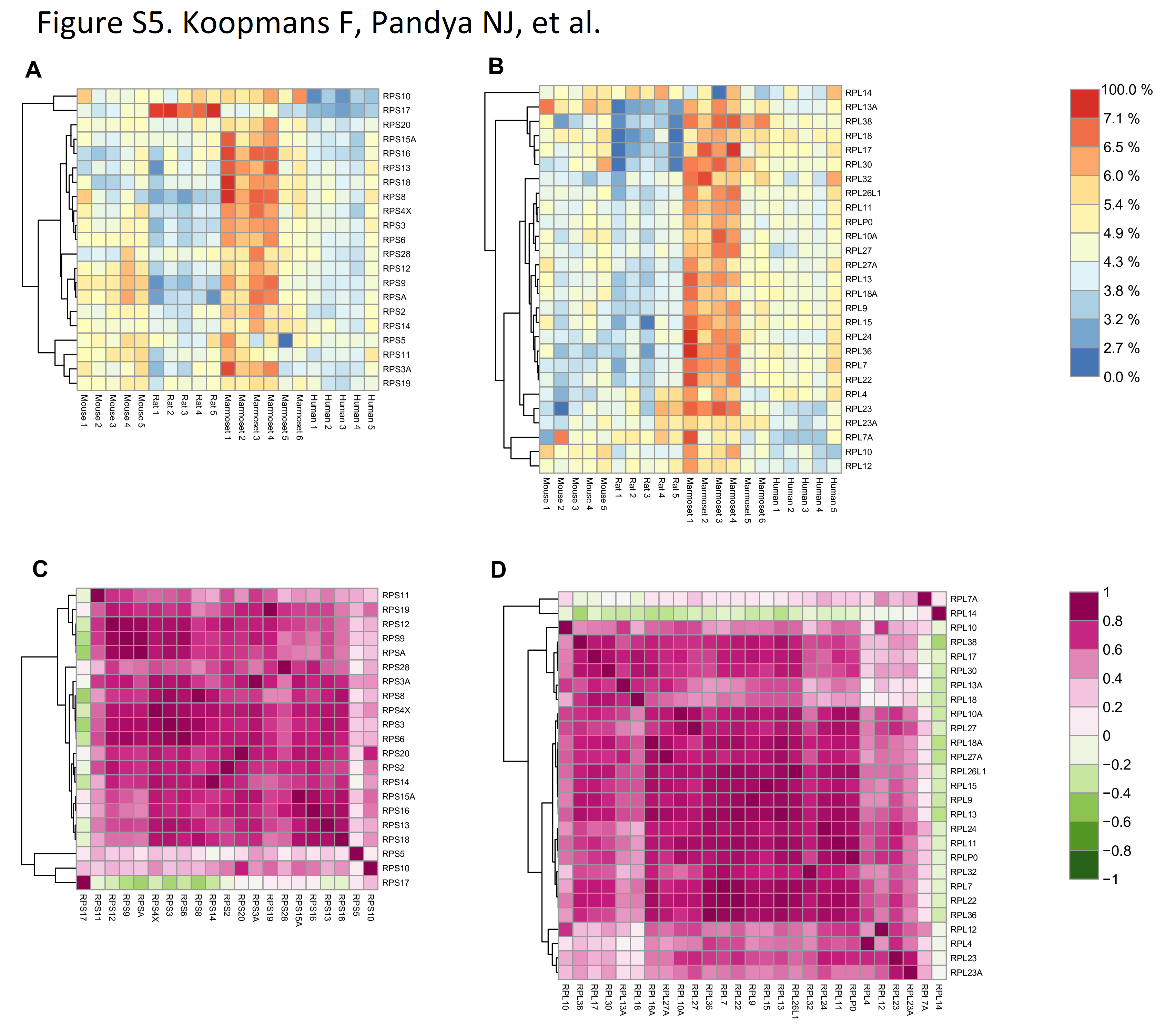

Supplement: FIGURE S5 — Quantified proteins in ribosomal protein complexes (top row) and the respective protein-protein Pearson correlation matrices (bottom row). Abundance values were scaled by their total over all samples to reveal their relative enrichment, if any, between species. The Pearson correlations were computed for pairs of proteins using their abundance values from all samples. Strong correlations are shown in dark purple while anti-correlations are shown in green, as visualized in the legend on the bottom-right. (A,C) small subunits of ribosomal proteins. (B,D) large subunits of ribosomal proteins. [file Image_5.TIFF]

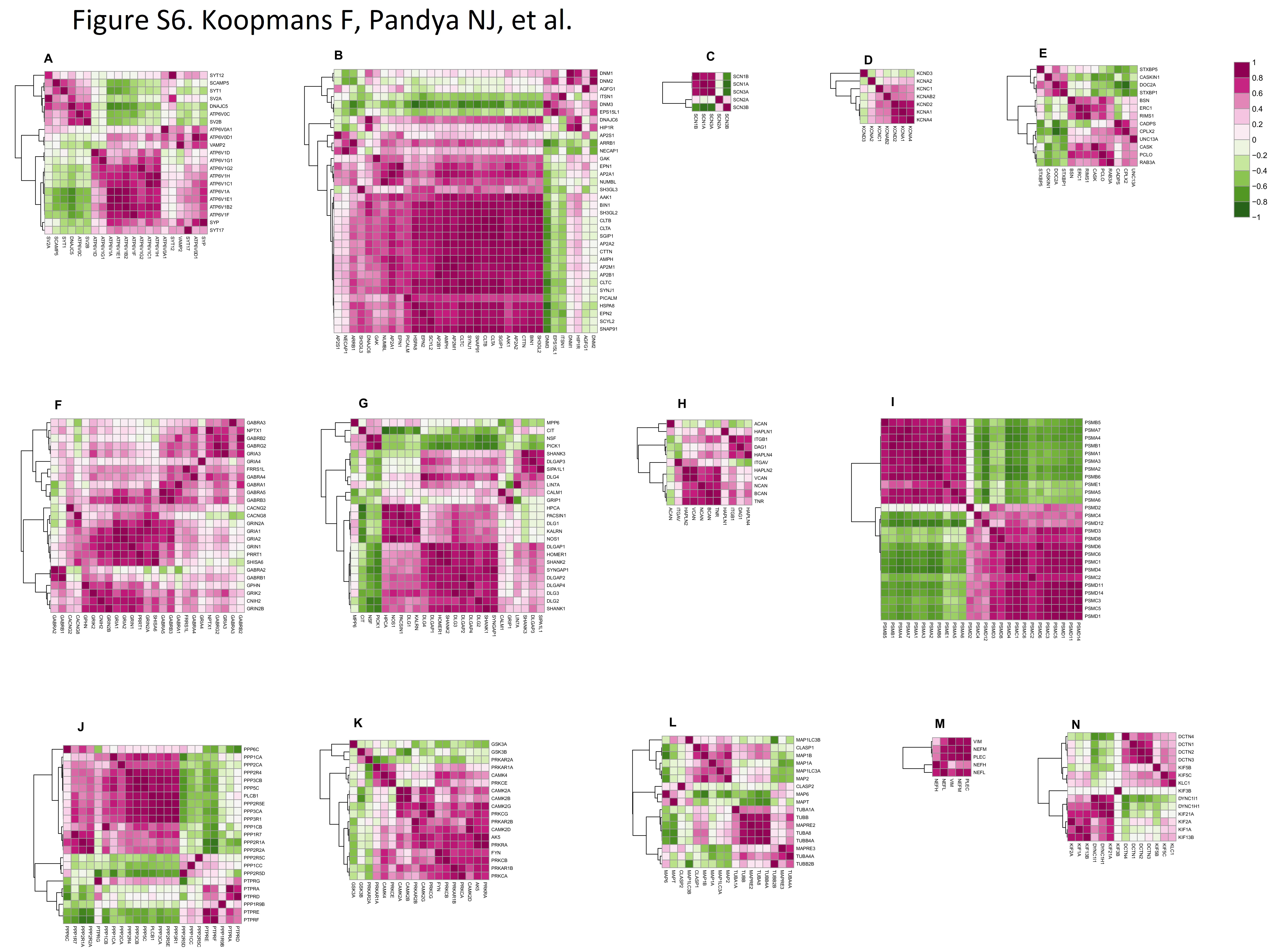

Supplement: FIGURE S6 — Correlation matrices for protein groups shown in Figure 3. The Pearson correlations were computed for pairs of proteins using their abundance values from all samples. Strong correlations are shown in dark purple while anti-correlations are shown in green, as visualized in the legend on the top-right. (A) Synaptic Vesicle. (B) Endocytosis. (C) Sodium channels. (D) Potassium channels. (E) Presynaptic scaffold. (F) Ligand-gated ion channels and associated proteins. (G) Postsynaptic density. (H) Extracellular matrix. (I) Proteasome. (J) Phosphatases. (K) Kinases. (L) Microtubules. (M) Neurofilaments. (N) Motor proteins. [file Image_6.TIFF]

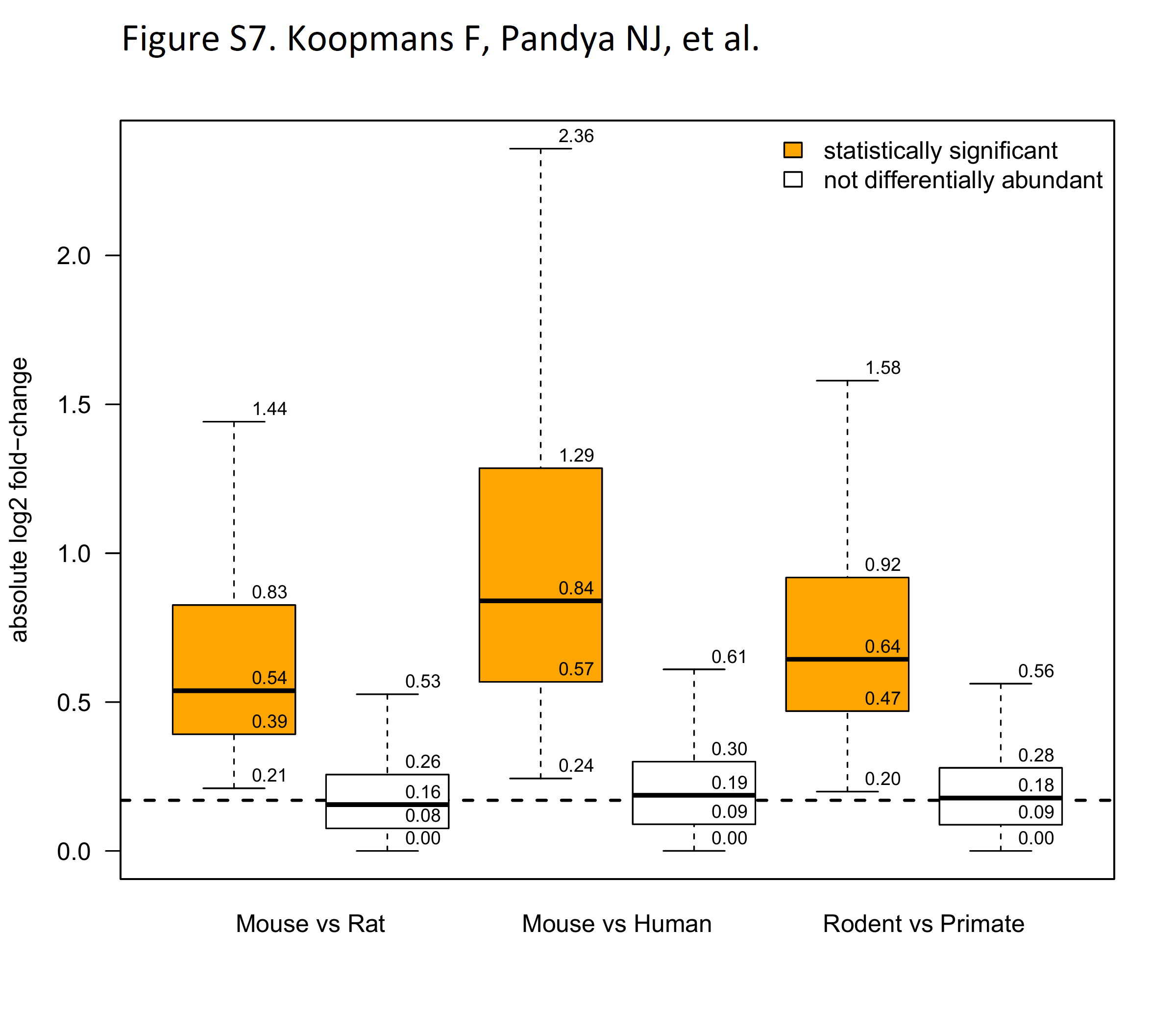

Supplement: FIGURE S7 — Protein abundance fold-changes for various species comparisons. The subset of differentially abundant proteins (at FDR adjusted p-value 0.005) is shown in orange while remaining proteins are shown in white. The median fold-change over unchanged proteins from all comparisons is 1.125 (0.17 on log2 scale), as visualized by a horizontal dashed line. [file Image_7.TIFF]
